# Correction: Ziranu et al. Navigating the Landscape of Liquid Biopsy in Colorectal Cancer: Current Insights and Future Directions. *Int. J. Mol. Sci. *2025, *26*, 7619

**DOI:** 10.3390/ijms27062535

**Published:** 2026-03-10

**Authors:** Pina Ziranu, Andrea Pretta, Giorgio Saba, Dario Spanu, Clelia Donisi, Paolo Albino Ferrari, Flaviana Cau, Alessandra Pia D’Agata, Monica Piras, Stefano Mariani, Marco Puzzoni, Valeria Pusceddu, Ferdinando Coghe, Gavino Faa, Mario Scartozzi

**Affiliations:** 1 Medical Oncology Unit, University Hospital and University of Cagliari, 09042 Cagliari, Italy; an.pretta@gmail.com (A.P.); sabagiorgio@live.it (G.S.); dario.spanu@gmail.com (D.S.); cleliadonisi@gmail.com (C.D.); alessandrapiadagata@gmail.com (A.P.D.); mariani.step@gmail.com (S.M.); marcopuzzoni@gmail.com (M.P.); valeria.pusce@gmail.com (V.P.); marioscartozzi@gmail.com (M.S.); 2 Division of Thoracic Surgery, Azienda di Rilievo Nazionale ed Alta Specializzazione “G. Brotzu”, 09121 Cagliari, Italy; paoloalb.ferrari@gmail.com; 3 Department of Medical Sciences and Public Health, University of Cagliari, 09042 Cagliari, Italy; flacau@tiscali.it (F.C.); monica.piras@unica.it (M.P.); gavinofaa@gmail.com (G.F.); 4 Clinical-Microbiological Laboratory, University Hospital of Cagliari, 09042 Cagliari, Italy; coghe.f@tiscali.it; 5 Department of Biology, College of Science and Technology, Temple University, Philadelphia, PA 19122, USA

**Error in Table and Legend**

In the original publication [1], there was a mistake in Table 5 and the legend for Table 5. The authors have added the following acronym: “GCP: comprehensive genomic profiling”, which was missing in the original version. Adjustments have also been made regarding the following:

- CRICKET study: changed from “50%” to “48%”.
- VELO trial: updated from “Phase II” to “Retrospective Analysis”.
- CAVE-2 GOIM: from “Panitumumab” to “Cetuximab + avelumab vs. cetuximab”; from “Comprehensive genomic profiling to identify resistance alterations” to “Baseline ctDNA CGP (FoundationOne Liquid CDx) in RAS/BRAFV600E-WT, MSS mCRC to guide rechallenge”; from “42% of patients showed resistance mutations; ultra-selection validated” to “41.7% had resistance mutations; 52.7% had actionable alterations (ESCAT); ultra-selection based on ctDNA may guide treatment”.

The correct legend and table appear below. 

**Table 5:** Overview of liquid biopsy studies in anti-EGFR therapy for metastatic colorectal cancer. EGFRi: epidermal growth factor receptor inhibitors; ctDNA: circulating tumor DNA; RAS-wt: RAS wild-type; PFS: progression-free survival; OS: overall survival; ORR: objective response rate; BRAF: B-Raf Proto-Oncogene, Serine/Threonine Kinase; MAP2K1: Mitogen-Activated Protein Kinase Kinase 1; VEGFi: Vascular Endothelial Growth Factor Inhibitors; PI3K: Phosphoinositide 3-Kinase; MET: Mesenchymal–Epithelial Transition Factor; BRAF/EGFR: B-Raf Proto-Oncogene, Serine/Threonine Kinase/Epidermal Growth Factor Receptor; GCP: comprehensive genomic profiling.

Study	EGFRi	Type of Study	Focus	Outcome	References
LIBImAb (ASCO 2023)	Cetuximab, Panitumumab	Phase III	ctDNA detection of RAS mutations in RAS-wt patients; 9.5% detected as RAS-mut	Refined patient stratification, potential shift to VEGFi for RAS-mut patients	[258]
FIRE-4	FOLFIRI + Cetuximab	Phase III	13% discordance between tissue and ctDNA RAS status	Highlighting importance of baseline ctDNA testing	[259,260]
Valentino Trial	Panitumumab + 5 fluorouracile	Retrospective Analysis	Identification of molecular alterations during panitumumab maintenance	Identification of poor PFS/OS predictors; ctDNA monitoring recommended	[261]
MODUL (NCT02291289)	Various EGFR inhibitors	Phase II/III	Adaptive therapy based on ctDNA dynamics	Pending results	-
COPERNIC (NCT05487248)	Chemotherapy + EGFR inhibitors	Phase II/III	Evaluating ctDNA-guided therapy in third-line setting	Pending results	-
CRICKET (Phase II)	Cetuximab + Irinotecan	Phase II	Rechallenge with cetuximab + irinotecan; 48% RAS mutations detected	Limited efficacy in RAS-mut patients; RAS-wt responded better	[262]
REMARRY	Panitumumab + Irinotecan	Retrospective Analysis	Rechallenge with panitumumab + irinotecan based on ctDNA RAS-wt reversion	Longer EGFRi-free interval improved outcomes; ctDNA crucial for selection	[263,264]
VELO Trial	Panitumumab + Trifluridine/Tipiracil	Retrospective Analysis	Rechallenge in RAS/BRAF-wt ctDNA patients	Prolonged benefit in ctDNA-confirmed RAS/BRAF-wt cases	[265]
CAVE Study	Cetuximab + Avelumab	Phase II	Combination of cetuximab and avelumab; non-ctDNA guided	OS benefit observed; non-ctDNA guided	[266]
CHRONOS	Panitumumab	Phase II	ctDNA-based ultra-selection for rechallenge with panitumumab	Confirmed value of ctDNA ultra-selection in rechallenge	[267]
REMARRY-PURSUIT	Panitumumab + Irinotecan	Phase II	Assessment of ctDNA RAS dynamics in rechallenge setting	Modest ORR (14%); exclusion of BRAF/EGFR mutations may have impacted outcomes	[263,264]
Mariani et al.	Cetuximab	Retrospective Analysis	Rechallenge strategy based on EGFRi-free interval and ctDNA RAS status	Improved outcomes with >14-month EGFRi-free interval	[268]
CAVE-2 GOIM (NCT05291156)	Cetuximab + avelumab vs. cetuximab	Phase II	Baseline ctDNA CGP (FoundationOne Liquid CDx) in RAS/BRAFV600E-WT, MSS mCRC to guide rechallenge	41.7% had resistance mutations; 52.7% had actionable alterations (ESCAT); ultra-selection based on ctDNA may guide treatment	[269]
Alshammari K et al.	Trametinib + Panitumumab	Phase II	Combination therapy in RAS, BRAF, MAP2K1 mutations; poor tolerability	Trial terminated due to poor tolerability	[270]
PERSPECTIVE	Tepotinib + Cetuximab	Phase II	Targeting MET amplification; limited efficacy	Accrual limitations; promising initial findings	[271]
PARERE (NCT04787341)	EGFR inhibitors	Phase II	Sequencing of pani->rego vs. rego->pani in ctDNA-selected RAS/BRAF wt mCRC	Panitumumab before regorafenib showed superior ORR; ctDNA-guided sequencing supports rechallenge strategy	[272]
C-PRECISE-01 (NCT04495621)	Cetuximab + PI3K inhibitor MEN1611	Phase Ib/II	Targeting PIK3CA mutations	Early phase; targeting actionable mutations via ctDNA	[273]
OrigAMI-1 (NCT05379595)	MET inhibitor Amivantamab	Phase II	Rechallenge in L-sided RAS/BRAF/ EGFR wt mCRC post-EGFRi; impact of EGFRi-free interval	Longer EGFRi-free interval (>8.8 mo) improved ORR (32% vs. 7%), PFS (7.0 vs. 2.8 mo), OS trend (16.1 vs. 10.4 mo)	[274]

**Missing Citation**

In the original publication [1], the number 272 was omitted at the end of paragraph 4.2.1 in relation to the PARERE study. This reference has now been included.

**Text Correction**

There was an error in the original publication [1]. The sentence describing the initial data for the CAVE-2 GOIM study has been corrected due to an error in the previous version. The incorrect sentence in the previous version is as follows: “The CAVE-2 GOIM trial, presented at ASCO GI 2025, employed comprehensive genomic profiling (CGP) via the FoundationOne Liquid CDx platform, identifying acquired RAS or BRAF mutations in 31.4% of patients and resistance alterations in 42%. Notably, patients who were wt for KRAS, NRAS, BRAFV600E, EGFR, ERBB2, MAP2K1, and PIK3CA experienced significantly longer PFS [269]”.

A correction has been made to Section 4, “Advanced Disease: Target and Response Evaluation in Metastatic Colorectal Cancer”, subsection 4.2.1, “Anti-EGFR Therapy: Treatment Selection, Resistance Mechanisms, and Rechallenge Strategies”, paragraph 11.

“A recent preplanned baseline translational analysis from the randomized phase II CAVE-2 GOIM trial, avelumab plus cetuximab versus cetuximab alone as anti-EGFR rechallenge in refractory ctDNA RAS/BRAFV600E wt mCRC patients, showed that baseline ctDNA CGP (FoundationOne Liquid CDx) identifies acquired RAS/BRAFV600 and non-RAS/BRAFV600 resistance alterations, supporting liquid-biopsy–guided hyper-selection for anti-EGFR rechallenge in refractory RAS/BRAFV600E–wild-type mCRC [269].”

In the first sentence of Section 4, “Advanced Disease: Target and Response Evaluation in Metastatic Colorectal Cancer”, subsection 4.2.1, “Anti-EGFR Therapy: Treatment Selection, Resistance Mechanisms, and Rechallenge Strategies”, paragraph 12. the word “Recently” has been corrected to the word “Moreover”.

**References**

Reference number 269 has been updated to include a recently published study related to CAVE-2 GOIM. Reference number 305 has been corrected to accurately reflect the molecular analyses of the BEACON study, as the previous version contained an error.

The authors state that the scientific conclusions are unaffected. This correction was approved by the Academic Editor. The original publication has also been updated.
